# Pregnant women’s knowledge of obstetric violence and related factors: baseline evidence from an implementation study in the central zone, Tanzania

**DOI:** 10.3389/fgwh.2025.1685292

**Published:** 2026-01-12

**Authors:** Theresia J. Masoi, Stephen M. Kibusi, Lilian Teddy Mselle, Nathanael Sirili

**Affiliations:** 1 Department of Clinical Nursing, The University of Dodoma, Dodoma, Tanzania; 2 Department of Public Health and Community Nursing, The University of Dodoma, Dodoma, Tanzania; 3 Department of Clinical Nursing, School of Nursing, Muhimbili University of Health and Allied Sciences, Dar es Salaam, Tanzania; 4 Department of Development Studies, School of Public Health and Social Sciences, Muhimbili University of Health and Allied Sciences, Dar es Salaam, Tanzania

**Keywords:** knowledge, mistreatment, obstetric violence, respectful care, Tanzania

## Abstract

**Background:**

Obstetric violence is globally acknowledged as a significant public health concern, with women in many countries reporting different types of mistreatment during pregnancy, childbirth, and the postpartum period, that negatively affect their physical and emotional health. Many women are vulnerable to obstetric violenceand without knowledge about their rights, do not identify specific manifestations of violence.

**Aim:**

This baseline study aimed to assess the current knowledge on obstetric violence in pregnant women and to determine possible associated factors in the central zone, Tanzania.

**Methods:**

The baseline cross-sectional study was conducted on 349 randomly selected pregnant women between February and April 2025. A standard semi-structured questionnaire in a kobo tool box software was used to collect data on knowledge of obstetric violence and associated factors. Both descriptive and inferential analyses were conducted using SPSS software version 29.

**Results:**

Overall, only 120(34.4%) of pregnant women had adequate knowledge of obstetric violence. The most commonly known components of obstetric violence were verbal 216(61.9%) and physical violence 211(60.5%). High levels of education, gravidity, and mobile phone usage for health-related issues were significantly associated with knowledge of obstetric violence. In addition, none of the pregnant woman reported being counselled about obstetric violence during their antenatal care.

**Conclusion:**

This baseline assessment offers essential information regarding the current state of knowledge about obstetric violence among pregnant women. The absence of reported counseling about obstetric violence during antenatal care highlights a critical gap. The results highlight the necessity for selective educational initiatives to enhance women's knowledge and their rights during pregnancy, childbirth and after childbirth in promoting respectful maternity care.

## Introduction

Globally, evidence of obstetric violence is reported and research shows that almost every woman goes through some level of disrespect and abuse during childbirth (1). The World Health Organization (WHO) defines obstetric violence (OV) as the control over a woman's body and reproductive processes through dehumanizing treatment that in turn negatively impacts her quality of life (2, 3). Obstetric violence (OV) is a widespread problem that affects women all over the world and is characterized by rude, violent, or careless treatment of women during pregnancy, childbirth and after childbirth. Physical assault, verbal abuse, unconsented medical procedures, refusal of pain relief, and systemic problems like a lack of privacy and autonomy are some examples of how this can show up (4, 5). Globally, the prevalence of OV during pregnancy and childbirth ranges from 15% to 99% with a prevalence of 44% in Sub-Saharan Africa (6, 7) and in Tanzania the prevalence ranges between 51.5 and 73.15 (3, 8).

The term “obstetric violence” in this study is used to refer to any act or treatment that occurs in the care provided during pregnancy, childbirth, and after birth, whether in health facilities or in community practices, that violates woman's dignity and rights and which undermines the quality of maternity care and safety of a woman (9).

OV is acknowledged by the World Health Organization (WHO) as a human rights violation and a major obstacle to attaining favorable health outcomes for mothers and newborns (2). Beyond just physical harm, OV can cause psychological trauma, a fear of childbirth, and a decrease in the use of maternal health care services (10). Obstetric violence contributes indirectly to maternal morbidity and mortality by deterring women from using recommended interventions in health care facilities (11). The literature reveals that violence can occur at the level of interaction between the woman and the provider, as well as through systemic failures at the health facility health system levels and at the community (12).

Obstetric violence is a type of gender-based violence that primarily affects women and is a result of unequal power dynamics in our society, and is recognised as both an indicator of poor healthcare practices and a key barrier to achieving improved maternal health outcomes (13). For instance, OV is classified as violence against women under national laws in nations like Argentina and Venezuela. According to a Brazilian study on women and gender in public and private settings, one in four women experience some form of violence during childbirth, including yelling, unpleasant procedures performed without their knowledge or consent, a lack of analgesia, and even carelessness (14).

The most mentioned risk factor in most of the literature is vaginal delivery compared to cesarean.

Section. This could be due to the longer time and interaction required during vaginal delivery as is a more prolonged and hands-on process than cesarean section. The longer time period and close contact might create more opportunities for inappropriate, disrespectful, or abusive behaviors referred to as obstetric violence (15). Other factors includes the number of Antenatal care contact (ANC) as mothers who had four or more ANC contacts were more likely to report obstetric violence than their counterparts, induction or augmentation of labor is reported to be the risk factors as the odds of experiencing obstetric violence is higher than those mothers who started labor spontaneously (16).

Obstetric violence has gained significant attention from the scientific community in recent years. Researchers from across the world continue to explore the meaning, driving factors, and measurement of obstetric violence during pregnancy and childbirth as to date there is no consensus at a global level on how it is defined and measured (17).

Understanding it requires considering the local context in which a study is conducted, as the meanings of respect, disrespect, and violence can vary across cultures, traditions, and locations. Cultural and geographical factors influence how obstetric violence is perceived, with some women either unaware of it or viewing it as a normal part of their experience (17).

In Tanzania, studies have identified several forms of obstetric violence, including physical violence, lack of supportive care and treatment, subjugation care, an unfavorable care environment, sexual violence, verbal violence, and emotional or psychological violence, these components were used in measuring knowledge level in the current study (18, 19).In addition we acknowledge Browser and Hill, and Bohren categorizations as commonly used frameworks in the field.

Throughout Tanzania obstetric violence is prevalent in its healthcare system. While the prevalence varies, D&A remains a significant concern. Individual and systemic factors influence both provider and patient perspectives of OV, providing a window into a complex and sensitive phenomenon. It is important that OV in Tanzania be viewed holistically, and that interventions target the multifaceted nature of the issue (3).

In most of the studies, women reported their experiences of birthing in simple terms of what they felt “good” and “bad”. Some women are found not to be aware of their rights during childbirth and consider their experience of mistreatment to be normal and sometimes may justify mistreatment as a way to assist them during childbirth (6). Women-related contributing factors to OV include a lack of knowledge on women's rights lack of autonomy and empowerment, and normalization of disrespect and abuse during childbirth by women, who think it is normal practice (4). The effectiveness of “obstetric violence” to challenge “normal” is dependent on how we understand “violence” and our collective ability to recognize it in the particular context due to cultural and geographical differences. On the other hand, many women, facing an event of greater vulnerability to OV and without knowledge about their rights, do not identify certain manifestations of violence (20).

This baseline study aimed to assess the current understanding of OV among pregnant women and identify the associated factors. The baseline assessment was conducted as part of the ongoing comparative interventional study (mHealth intervention) aimed at looking the crucial insights into the existing knowledge gaps and informs the design and implementation of subsequent study phases.

## Methods and materials

### Study setting

The current study was conducted in Dodoma and Singida regions in central Tanzania. These are among the regions with high maternal mortality rates in the country (21). One contributing factor to the high maternal mortality rate is home deliveries, which stands at 35.5% in Dodoma and 40% in Singida, mainly due to fear of mistreatment and violence during pregnancy, labor and after delivery (22, 23). In addition, these regions have a high number of deliveries. For example, in 2022, Dodoma Municipal Council recorded approximately 25,484 deliveries while Singida Municipal recorded 10,380 deliveries (24).

### Study design

The baseline assessment, described in this study, involved an analytical cross-sectional study administered to pregnant women prior to being enrolled in the m-Health intervention study. The primary objective of the m-Health intervention was to assess the incidence of obstetric violence as reported by pregnant women and providers. Pregnant women at the GA of 32 weeks were recruited to the study (both control and intervention groups) and followed until six weeks after delivery. At the time of enrolment, pregnant women in the control and intervention groups completed a questionnaire to assess their baseline level of knowledge about obstetric violence. Thereafter, both groups were trained on the contextual components of OV and what is required from them during the follow up period. Then six weeks post-delivery, both groups will again fill the same questionnaire to assess for interim change in the level of knowledge after the follow up period as well as assessing obstetric violence experience/incidences.

### Study population and sample size estimation

A total of 349 pregnant women at 32 weeks of gestation and who will be available throughout the study period and own a mobile phone were randomly selected in the implementation study (m-Health). Pregnant women were selected by a systematic random method in which every third pregnant woman among those who met the inclusion criteria and consented to participate in the study and available during the study period, was selected. The sampling frame were pregnant women attending the ANC visits with the gestation age of 32 weeks.

The sample size (Pregnant women) was obtained by using the following formula;

(25) $$n=2\left\{z\alpha \sqrt{\left[\pi 0(1-\pi 0)\right]}+Z\beta \sqrt{\left[\pi 1(1-\pi 1)\right]}\right\}/(\pi 1-\pi 0)2$$

The values for π0 and π1 was taken from a study that was done on Community and health system intervention to reduce disrespect and abuse during childbirth in Tanga Region, Tanzania: A comparative before-and-after study. In which the baseline proportional of disrespect and abuse was 13.10% and after the intervention was 3.2% (26).The ratio of the Intervention group to control was 1:1, Figure 1 below shows the sampling and intervention flow chart.

**Figure 1:**
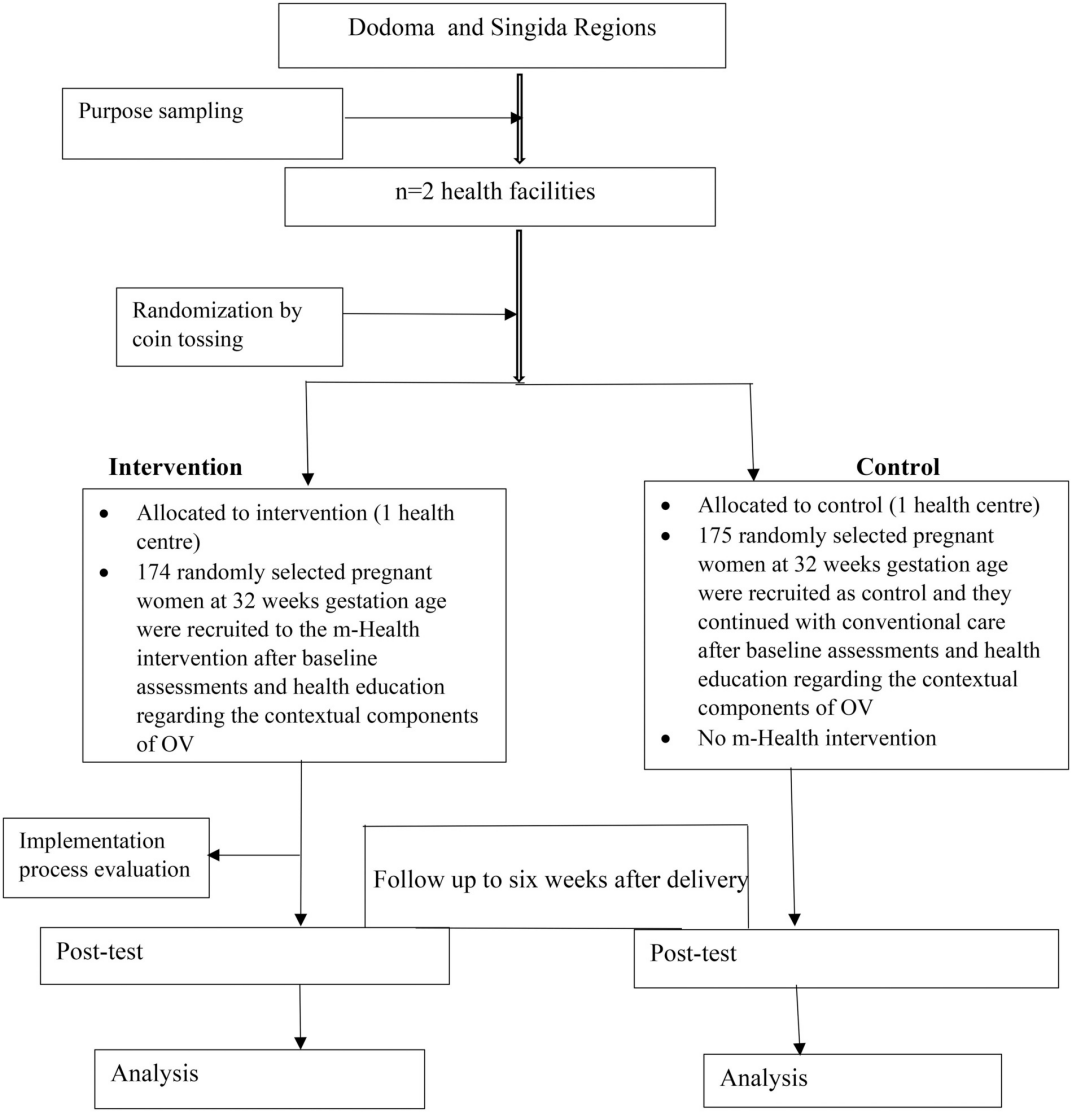
Sampling and m-health intervention flow chart.

### Study variables and measurement

Dependent variables: knowledge about OV was categorised as follows: Those unable to mention any of the OV components were considered to have no knowledge. Those respondents who mentioned one to two OV components were considered to have little knowledge. Women who mentioned three to five OV components were considered to have good knowledge, and the respondent was considered to have very good knowledge if she mentioned six or more OV components. For further analysis, the four categories were re-grouped into two categories of adequate and inadequate knowledge. This method of scoring was adopted and modified from the study done in Brazil (14).

Independent variables: Independent variables included occupation, age, parity, education level, marital status, mobile phone usage and health insurance use, gravidity.

### Data collection tools and methods

Data were collected at the antenatal care (ANC) clinics at the two selected health care facilities between February and April, 2025. Women attending routine ANC appointments were consecutively approached by trained research assistants in the clinic waiting area. Eligibility was assessed according to the study inclusion criteria (gestation of 32 weeks, available throughout the study period and own a mobile phone). Eligible women received verbal and written information about the study and provided written informed consent in a private setting before any data collection. Face-to-face interviewers administered semi-structured questionnaires with both closed and open-ended questions was used. Initially, the questionnaire was created in English and subsequently translated into Kiswahili, which is Tanzania's national language and the primary language of the study participants. The questionnaire covered various aspects of the study, including socio-demographic information, life styles, Partner characteristics, Obstetric history and knowledge of obstetric violence. Four data collectors were recruited and trained on the purpose of the study, ethics, and the data collection tool. Two data collectors collected data in Dodoma and two in Singida. Interviews were conducted face-to-face in a private room adjacent to the ANC clinic to ensure confidentiality and to reduce interruptions. The questionnaire was uploaded to kobo Toolbox software, and tablets were used for data collection.

### Pre-testing of the tool

To ensure the validity and reliability of the tool used to measure knowledge of obstetric violence among pregnant women, the data collection instrument was pre-tested on 10% of the calculated sample size, which equalled 35 pregnant women, prior to the actual data collection. The pre-test data were analyzed using SPSS version 29. The findings informed both Principal Components Analysis (PCA) and scale reliability analysis. For PCA, the threshold for factor loading was set at 0.50, as recommended by previous scholars (27). Following PCA, all items loaded in one component, implying that they were unidimensional; hence, they were subjected to scale analysis to assess the reliability of the tool using Cronbach's alpha, with a cut-off value of ≥0.70 considered acceptable. In this study, Cronbach's alpha was 0.868, indicating high internal consistency. The tool was subsequently reviewed and revised to ensure it captured valid and reliable information and to improve clarity and understanding.

### Data analysis

Data from the KoboToolbox software were retrieved and transferred to the Statistical Product for Service Solutions (SPSS) software version 29. Before conducting the analysis, data cleaning was performed using frequency distribution tables to ensure data accuracy.

Descriptive analysis was used to analyse participants’ demographic and obstetric characteristics to determine the frequencies and a percentages of their distributions, as well as the mean and standard deviation of knowledge scores. Overall scores of participants with adequate and inadequate knowledge of obstetric violence were also calculated. The chi-square test was used to establish the relationship between the categorical variables. Since the chi-square test does not account for simultaneous relationship between dependent variable and a set of independent variables, a binary logistic regression was employed to ascertain the relationship between knowledge level of obstetric violence and the associated factors. The selection of the binary logistic regression model is based on the fact that the knowledge level was categorised into two dichotomous categories. Before the multivariate regression analysis was done, the bivariate analysis was done on the outcome variable with each of the independent/predictor variables to get an unadjusted/crude odds ratio (COR). The variables which were statistically significant at 95% CI were included in the multivariate regression analysis.

### Ethics approval and consent to participate

Ethical clearance for this study was obtained from the Senate Research and Ethics Committee of the Muhimbili University of Health and Allied Sciences in Tanzania (Ref. No. DA. 282/298/01.C/1758). Permission for data collection was obtained from the Ministry of Health, the Ministry responsible for local government, regional and district authorities, Heads of health facilities. Written informed consent was obtained individually from each study participant before the commencement of the interview. The objective and importance of the study were explained to participants. Participants were encouraged to communicate freely, as their information would only be used for research purposes. They were further informed that their participation was purely voluntary and they could withdraw from the interview at any time they wished to do so. Confidentiality of the information shared was also ensured.

## Results

- Social demographic and obstetric characteristics of the participants

This study involved 349 pregnant women. The most prominent age group, 82.5% (*n* = 288), ranged between 20 and 34 years. The majority of the participants, 41.8% (*n* = 146) had a secondary level of education, and a few had college/university education, 19.8% (*n* = 69). Additionally, the majority of the study participants, 70.8% (*n* = 247), were self-employed. Furthermore, the study also explored Obstetric characteristics among the study participants. Out of 349 participants, 59.9% (*n* = 209) had two to four pregnancies. Moreover, only 21% (*n* = 73) started their first Antenatal visit between 1 and 12 weeks as recommended. Additionally, none of the participants reported being counselled about obstetric violence during antenatal care (Table 1).

ii. Overall knowledge scores and Scores of knowledge on the specific obstetric violence components

**Table 1:** Social demographic and obstetric characteristics of the pregnant women (*n* = 349).

Variable		*n*	%
Age	<20	16	4.6
	20–34	288	82.5
	≥35	45	12.9
Marital status	Not married	88	25.2
	Married	261	74.8
Education level	Primary	134	38.4
	Secondary	146	41.8
	Colleges/universities	69	19.8
Occupation status	Self-employed	247	70.8
	Not-employed	80	22.9
	Employed	22	6.3
Mobile phone usage for health-related issues	No	283	81.6
	Yes	64	18.4
Alcohol usage	I don't use	337	96.6
	I use it once in a while	12	3.4
	I use it every day	0	0
Smoking/previously smoked	No	347	99.4
	Yes	2	0.6
Health insurance usage	No	324	92.8
	Yes	25	7.2
Live with partner/husband	No	7	2.7
	Yes	254	97.3
Gravidity	1	88	25.2
	2–4	209	59.9
	5+	52	14.9
Gestation age	32 weeks	113	32.4
	33 weeks	92	26.4
	34 weeks	110	31.5
	35 weeks	34	9.7
Gestation age at first antenatal visit	1–12 weeks	73	21.0
	13–26 weeks	259	74.4
	27–40 weeks	16	4.6
Parity	0	96	27.5
	1	77	22.1
	2–4	163	46.7
	5+	13	3.7
Counseled about obstetric violence during antenatal care	No	349	100
	Yes	0	0

Overall knowledge level of obstetric violence was assessed, and it was categorized as “Inadequate Knowledge” or “Adequate Knowledge in the end analysis.” Findings showed that only 120 (34.4%) of pregnant women had adequate knowledge of obstetric violence. Additionally, knowledge level assessment on the specific obstetric violence components was also conducted. The most known obstetric violence components were verbal violence in the sub-category of poor communication pattern 216 (61.9%) followed by physical violence in the sub-category of violent physical act during labor and delivery 211(60.5%). The least known component was sexual violence in the sub category of husband stitches 20(5.7%) (Table 2).

iii. The Association Between Sociodemographic and Obstetric Characteristics and Knowledge of Obstetric Violence

**Table 2:** Scores of knowledge on obstetric violence specific components (*n* = 349).

Aspects of OV	Yes	I don't know	Total
	*n* (%)	*n* (%)	*n* (%)
Physical violence	159 (45.6)	190 (54.4)	349 (100)
Violent physical act during labor and delivery	211 (60.5)	138 (39.5)	349 (100)
Painful routine procedures that lacks evidence but are done by experience	95 (27.2)	254 (72.8)	349 (100)
Physical violence related to traditional practices during Pregnancy, childbirth and after childbirth	104 (29.8)	245 (70.2)	349 (100)
Lack of supportive care	102 (29.2)	247 (70.8)	349 (100)
Denying care and treatment	129 (37)	220 (63)	349 (100)
Delayed care and treatment	92 (26.4)	257 (73.6)	349 (100)
Neglect/abandonment	160 (45.8)	189 (54.2)	349 (100)
Subjugation	153 (43.8)	196 (56.2)	349 (100)
Loss of control of what is happening during pregnancy, childbirth and after childbirth	97 (27.8)	252 (72.2)	349 (100)
Infantilization	63 (18.1)	286 (81.9)	349 (100)
Objectification	83 (23.8)	266 (76.2)	349 (100)
Lack of information of labor progress and procedure	102 (29.2)	247 (70.8)	349 (100)
Repeated procedures and treatment without consent	132 (37.8)	217 (62.2)	349 (100)
Forced treatment and procedure	78 (22.3)	271 (77.7)	349 (100)
Unfavourable care environment	69 (19.8)	280 (80.2)	349 (100)
Facility culture and Lack of policies	131 (37.5)	218 (62.5)	349 (100)
Lack of privacy and confidentiality	59 (16.9)	290 (83.1)	349 (100)
Lack of resources	108 (30.9)	241 (69.1)	349 (100)
Sexual Violence	164 (47)	185 (53)	349 (100)
Inappropriate sexual behavior	92 (26.4)	257 (73.6)	349 (100)
Husband stitches	20 (5.7)	329 (94.3)	349 (100)
Inappropriate sexual practice	135 (38.7)	214 (61.3)	349 (100)
Verbal Violence	205 (58.7)	144 (41.3)	349 (100)
Poor communication pattern	216 (61.9)	133 (38.1)	349 (100)
Unfriendly language and comments from partner and relatives	163 (46.7)	186 (53.3)	349 (100)
Psychological Violence	123 (35.2)	226 (64.8)	349 (100)
Blaming a woman for child birth poor outcome	88 (25.2)	261 (74.8)	349 (100)
Detention of a woman and her newborn to the health facility	57 (16.3)	292 (83.7)	349 (100)
Stigma and discrimination	165 (47.3)	184 (52.7)	349 (100)
Judgmental care	69 (19.8)	280 (80.2)	349 (100)

The analysis identified several factors that significantly influence the knowledge levels of obstetric violence among pregnant women as determined by chi-square tests. Women with college or university education were i knowledgeable (35%, *p* < 0.001) compared to those with primary level of education. Pregnant women who use mobile phones for health-related issues showed more knowledge (30.3%, *p* < 0.001) on obstetric violence compared to their counterparts. Furthermore, 21.7% of those with adequate knowledge had five or more pregnancies. Similarly, 72.5% of women with adequate knowledge had a parity between 2 and 4 (*p* < 0.001 for both gravidity and parity). This indicates that repeated exposure to pregnancy and childbirth enhances knowledge. Other factors such as age, marital status, and occupation showed no significant association with knowledge level (*p* > 0.05), indicating these factors do not directly influence knowledge. Notably, no participants reported having been counselled about obstetric violence during antenatal care, revealing a critical gap in current healthcare practices (Table 3).

iv. Factors associated with knowledge of obstetric violence

**Table 3:** Association between demographic and obstetric characteristics and knowledge of obstetric violence (*n* = 349).

Variable		Inadequate knowledge		Adequate knowledge		Chi-square *p*-value
		*n*	%	*n*	%	
Region	Dodoma	105	45.9	68	56.7	3.68
	Singida	124	54.1	52	43.3	0.06
Age	<20	12	5.2	4	3.3	0.67
	20–34	188	82.1	100	83.3	0.72
	≥ 35	29	12.7	16	13.3	
Marital status	Not married	60	26.2	28	23.3	0.34
	Married	169	73.8	92	76.7	0.56
Education level	Primary	102	44.5	32	26.7	28.54
	Secondary	100	43.7	46	38.3	<0.001*
	Colleges/universities	27	11.8	42	35	
Occupation	Self-employed	168	73.4	79	65.8	2.50
	Not-employed	49	21.4	31	25.8	0.29
	Employed	12	5.2	10	8.3	
Mobile phone use for health-related issues	No	200	87.7	83	69.7	16.79
	Yes	28	12.3	36	30.3	.000*
Health insurance usage	No	216	94.3	108	90	2.21
	Yes	13	5.7	12	10	0.14
Live with partner/husband	No	3	1.8	4	4.3	1.51
	Yes	166	98.2	88	95.7	0.22
Education level of the partner/husband	Primary	54	32	20	21.7	4.12
	Secondary	74	43.8	41	44.6	0.13
	Colleges/universities	41	24.3	31	33.7	
Different in education level from the partner	My partner's education level is higher than mine	73	43.2	27	29.3	4.85
	My education level is higher than that of my husband	20	11.8	13	14.1	0.09
	Our education level is similar	76	45	52	56.5	
Spouse/partner occupation	Self-employed	118	69.8	64	69.6	0.07
	Non-employed	24	14.2	14	15.2	0.97
	Employed	27	16	14	15.2	
Age differences with the partner	The wife is older than the husband	4	2.4	1	1.1	2.13
	We are the same age	10	5.9	4	4.3	.711,c
	Wife is younger than the husband by 1–4	109	64.5	66	71.7	
	Wife is younger to the husband by 5–9	45	26.6	21	22.8	
	Husband is older by 10 or more years	1	0.6	0	0	
	Wife is older by 10 or more	0	0	0	0	
Gravidty	1	82	35.8	6	5	40.78
	2–4	121	52.8	88	73.3	<0.001*
	5+	26	11.4	26	21.7	
Gestation age	32 weeks	81	35.4	32	26.7	4.41
	33 weeks	59	25.8	33	27.5	0.22
	34 weeks	71	31	39	32.5	
	35 weeks	18	7.9	16	13.3	
Gestation age at first antenatal visit	1–12	41	17.9	32	26.9	4.15
	13–26	176	76.9	83	69.7	0.13
	27–40	12	5.2	4	3.4	
Parity	0	84	36.7	12	10	51.30
	1	59	25.8	18	15	<0.001*
	2–4	76	33.2	87	72.5	
	5+	10	4.4	3	2.5	
Counselling about obstetric violence during antenatal care	No	229	100	120	100	
	Yes	0	0	0	0	

The symbol “*” showing the emphasis that the *p*-value is significant.

Logistic regression was performed to assess the association between the selected variables and knowledge on obstetric violence, controlling for potential confounders. Parity was excluded in multiple regression, due to multicollinearity with gravidity as both measure aspects of reproductive history and are highly correlated [The results reveal that the variables parity and gravidity have notably high VIF values (19.98 and 20.24, respectively) accompanied by very low tolerance levels of 0.05]. Additionally, Findings shows that women in Dodoma were 1.71 times more likely to have adequate knowledge on obstetric violence compared to those in Singida (*p* = 0.04), becoming significant after adjustment. Furthermore women with college/university education were nearly eight times to have adequate knowledge (OR = 7.99, *p* < 0.001) compared to those with primary education. Women with 2–4 pregnancies were about 17 times more likely to have higher knowledge compared to first-time mothers (OR = 16.78, *p* < 0.001), and those with 5 + pregnancies were over 25 times likely to have adequate knowledge (OR = 25.61, *p* < 0.001) suggesting that increased reproductive experience is strongly associated with greater knowledge (Table 4).

**Table 4:** Results of logistic regression model for a factor associated with knowledge on obstetric violence (*n* = 349).

Variables	Crude OR	95% C.I	*P*-value	Adjusted OR	95% C.I	*P*-value	Collinearity statistic	
		Lower-upper			Lower-upper		Tolerance	VIF
Region							0.99	1.01
Dodoma	1.54	0.99–2.41	0.06	1.71	1.02–2.87	0.04		
Singida	Ref			Ref				
Education level							0.83	1.20
Primary	Ref			Ref				
Secondary	1.47	0.86–2.49	0.16	2.04	1.14–3.66	0.02		
College/University	4.96	2.65–9.27	<0.001	7.99	3.62–17.66	<0.001		
Mobile phone usage for health-related issues							0.86	1.16
No	Ref			Ref				
Yes	3.10	1.78–5.40	<0.001	1.92	0.97–3.79	0.06		
Gravidity							0.05	20.24
1	Ref			Ref				
2–4	9.94	4.15–23.80	<0.001	16.78	6.40–43.98	<0.001		
5+	13.67	5.07- 36.83	<0.001	25.61	8.50–77.17	<0.001		
Parity							0.05	19.98
0	Ref							
1	2.136	0.957–4.76	0.06					
2–4	8.013	4.065–15.796	<0.001					
5+	2.1	0.505–8.731	0.31					

## Discussion

This study describes a baseline assessment aimed at understanding pregnant women's knowledge of obstetrics. This baseline assessment seeks to address this gap by evaluating the current level of knowledge and identifying factors that may influence their understanding of OV. Understanding the factors influencing knowledge of obstetric violence is critical for developing interventions to enhance awareness and promote respectful maternity care.

The baseline assessment revealed that overall knowledge of OV among participants was limited. Only 34.4% of participants had adequate knowledge. Women were more aware of some t forms of OV, such as physical and verbal abuse compared to other forms such as sexual violence in the subcategory of husband stitches. Many participants were unfamiliar with their rights during childbirth, including the right to informed consent and the right to refuse medical interventions. For example, only 29.2% knew about the right to be given information about their care. This finding aligns with a study on the sense of birth, which also found that women's knowledge of obstetric violence was low (14).

Studies in Tanzania suggest that women's knowledge of obstetric violence is influenced by contextual factors such as limited health literacy, normalization of abusive practices, and lack of empowerment to demand respectful care. Fear of mistreatment and limited awareness of womens’ rights, cultural beliefs and un equal gender norms constrained their ability to question or report abusive practices (10, 28).

The current study showed higher education levels to be strongly associated with adequate knowledge of obstetric violence. Women with college/university education have nearly eight times more knowledgeable compared to those with primary education. This indicates that education increases exposure to information and critical thinking abilities, which makes women more aware and knowledgeable about obstetric violence. These findings aligns with broader research indicating that socio-economic factors, such as education, influence health-related knowledge and awareness (29, 30).

Furthermore, the current study also found that women with multiple pregnancies (2–4 or 5 + pregnancies) have significantly adequate knowledge compared to those with one pregnancy. This may reflect increased exposure to antenatal care and healthcare interactions, where women may learn about obstetric violence through formal education or personal experiences. Women with more pregnancies likely have more opportunities to engage with healthcare providers, increasing their awareness. This finding is not different from other studies that found multiparous women may be more likely to share experiences and information, creating a supportive network where knowledge about obstetric violence can be shared and discussed (31).

The current study also found that using mobile phones for health-related issues is associated with adequate knowledge (OR = 1.92, *p* = 0.06), though this is marginally non-significant in the adjusted model. This suggests that mobile phones may serve as an effective tool for disseminating health information, particularly in resource-limited settings. The marginal significance could indicate confounding by other factors, such as education, but still highlights the potential of mobile technology.Similar findings have been reported by the World Health Organization and other studies, that mobile phone usage can potentially be a valuable tool for health education and information access hence health behavior change (32, 33).

No respondents reported receiving counseling about obstetric violence during antenatal care highlighting a critical gap that need to be worked at, as it hinders women of information regarding their right.

Generally, the findings of this baseline assessment highlight the significant knowledge gaps regarding OV among pregnant women. The limited understanding of OV, its various forms, and women's rights during childbirth underscores the need for targeted interventions to improve awareness and empower women to advocate for respectful maternity care. These findings reflect the need for community-based awareness and facility-level interventions that address both knowledge gaps and structural barriers within the Tanzanian maternity care system. The identified factors associated with knowledge of OV provide valuable insights for designing effective interventions.

## Strengths and limitations

The study examines the critical issue of obstetric violence, especially from the perspective of pregnant women. This contributes to ongoing efforts to promote respectful maternity care in Tanzania. Focusing on knowledge and awareness provides valuable insights into maternal health.

Conversely, the present study may have faced the following limitations: data on knowledge and related factors were self-reported, which could introduce recall bias or social desirability bias, especially on sensitive topics like violence. Using anonymous questionnaires and training interviewers helped reduce these issues, and participants were encouraged to speak openly. Although two regions (urban and rural) were included, the findings may not represent all regions in Tanzania, particularly rural areas. Expanding the study to include more regions with diverse sociocultural contexts, especially rural ones, would enhance its generalizability.

### Theoretical and practical implications

The current study provides crucial insights into the existing knowledge gaps and informs the design and implementation of subsequent studies. The results highlight the need for targeted educational programs and interventions to improve pregnant women's understanding of their rights and empower them to advocate for respectful maternity care. The limited understanding of OV, its various forms, and women's rights during childbirth underscores the need for targeted interventions to improve awareness and empower women. The identified factors associated with knowledge of OV provide valuable insights for designing effective interventions.

## Conclusion

This baseline assessment offers essential information regarding the current state of knowledge about OV among pregnant women. The current study identifies education level, gravidity, and mobile phone use as significant factors in the understanding of obstetric violence among pregnant women in Tanzania. Higher education and repeated pregnancies significantly boost the chances of sufficient knowledge. Mobile phones emerge as a potential health education tool. The results highlight the necessity for selective educational initiatives to enhance women's knowledge about OV, their rights during labor, and the provision of respectful maternity care, especially for less-educated women and first-time mothers, and the inclusion of obstetric violence education in antenatal care. Harnessing mobile technology could also increase awareness, support actions towards the prevention of obstetric violence, and promote respectful maternity care. Further studies should aim at developing and testing culturally sensitive interventions that respond to the unique needs of diverse populations and facilitate the adoption of respectful maternity care practices.

## Data Availability

The original contributions presented in the study are included in the article/Supplementary Material, further inquiries can be directed to the corresponding author.
